# Radiographic Assessment of Bilateral Asymmetry in the Upper Extremities of Living Humans

**DOI:** 10.7759/cureus.35957

**Published:** 2023-03-09

**Authors:** Abdurrahman F Kharbat, Cameron T Cox, Jarrod M Martinez, Brendan J MacKay

**Affiliations:** 1 Neurological Surgery, Texas Tech University Health Sciences Center, Amarillo, USA; 2 Orthopedic Surgery, Texas Tech University Health Sciences Center, Lubbock, USA

**Keywords:** humerus length, ulnar length, ulnar shortening, radius shortening, bone asymmetry, bilateral asymmetry, upper extremity asymmetry

## Abstract

Objective

Injuries resulting from trauma or tumor resection may cause length alterations in the bones of the upper extremities (UE) requiring reconstruction. Direct contralateral bone is often used to determine the appropriate length for reconstruction but fails to account for potential asymmetry. Given the paucity of data assessing asymmetry in living populations and the need for accurate length approximation, we developed a study evaluating UE long bone asymmetry using radiographic imaging in living subjects.

Methods

Bilateral X-ray images previously obtained for traumatic injury or chronic osseous conditions were retrospectively collected for adult subjects (ages 18-81). After screening, 61 patients met the inclusion criteria: 28 radii, 29 ulnae, and 19 humeri. Three serial measurements were taken, and the median was used for subsequent analysis. Wilcoxon signed-rank tests were performed to assess differences in contralateral bone lengths. Bootstrapping was utilized to obtain sample sizes of 200, 500, and 1000 for each bone.

Results

The difference in mean absolute length was 27.0 mm for the humerus, 8.6 mm for the radius, and 7.5 mm for the ulna. Neither the left side nor the right side was significantly longer for any bone. In 57.9% (11/19) of patients, the right humerus was longer than the left; in 60.7% (17/28), the right radius was longer than the left; and in 48.3% (14/29), the right ulna was longer than the left. All other measurements showed the left was longer than the right.

Wilcoxon signed-rank tests did not find significant differences between contralateral pairs in any direct measurement group. In bootstrap samples, significant differences in length (p ≤ 0.05) were seen in all samples (n = 200, 500, and 1000) for both humerus and radius but only the 1000 sample group for the ulna.

Conclusions

Direct contralateral measure may be an appropriate method of length estimation for the humerus, radius, and ulna in post-industrial humans.

## Introduction

The presence of bilateral variations in upper and lower limb bone lengths has historically been attributed to differential mechanical stress and strain placed upon the limb bones during periods of bone growth over the lifespan of an individual [1]. In the upper extremity (UE), the bones are typically longer on the right side (most often the dominant hand), whereas greater length is observed on the left side in the lower extremity (LE) [1]. It has been suggested that the presence of bilateral bone length asymmetry in the upper and lower extremities, which is known as “cross-symmetry,” is due to contralateral muscle contractions that serve to support the musculoskeletal system during periods of bone growth [1,2].

One study examined a large sample of non-adult skeletal remains from English archeological sites, confirming that bone length asymmetry is established during growth and development [3]. In this study, infants and young children exhibited no significant asymmetry, while older children and adolescents exhibited right-sided upper extremity asymmetry [3]. This indicates that biomechanical forces strongly influence the development of bilateral upper extremity asymmetry through a natural side-dominant process [3]. The development of asymmetry through childhood is postulated to be a function of the differential forces placed on the appendages via physical activity. Bilateral asymmetry was also found in diaphyseal and epicondylar breadths of the UE bones in adolescents and young adults but not infants [3].

Length alterations of the upper extremity bones have been linked to significant changes in function and pain; however, there is no consensus regarding the degree of length alteration, which constitutes pathological functional changes [4-6]. Congenital defects, tumor resection, or traumatic injury may cause length alterations and/or segmental bone defects that may require reconstruction. In order to achieve the maximum function of the affected limb, physicians must make treatment decisions regarding the appropriate length to optimize outcomes in these populations [7-9]. Thus, it is important to understand the degree of normal, non-pathological bilateral asymmetry in post-industrial humans as it may influence decisions regarding operative versus nonoperative treatment. When surgery is indicated, contralateral bone length is often used to determine the target length for reconstruction, yet it fails to account for bilateral asymmetry.

Our understanding of these musculoskeletal asymmetries remains limited, as previous studies have examined skeletal remains or cadavers rather than living patients. Numerous cadaver studies have investigated the relationship between length alterations in the UE long bones and functional deficits (e.g., biomechanical models showing ulnar shortening effects on distal radioulnar joint pressure and stability) [10,11]. However, the effects of length alterations seen in cadaveric models cannot fully demonstrate the potential functional limitations and/or pain experienced by living patients, and studies of forearm-shortening and forearm-lengthening procedures have reported significant changes in pain and range of motion [5,6]. Given the paucity of data addressing asymmetry in living subjects and the importance of accurate length estimates, we designed the present study to evaluate the asymmetry of the humerus, radius, and ulna by retrospectively analyzing radiographic images of living subjects.

## Materials and methods

**Figure 1:**
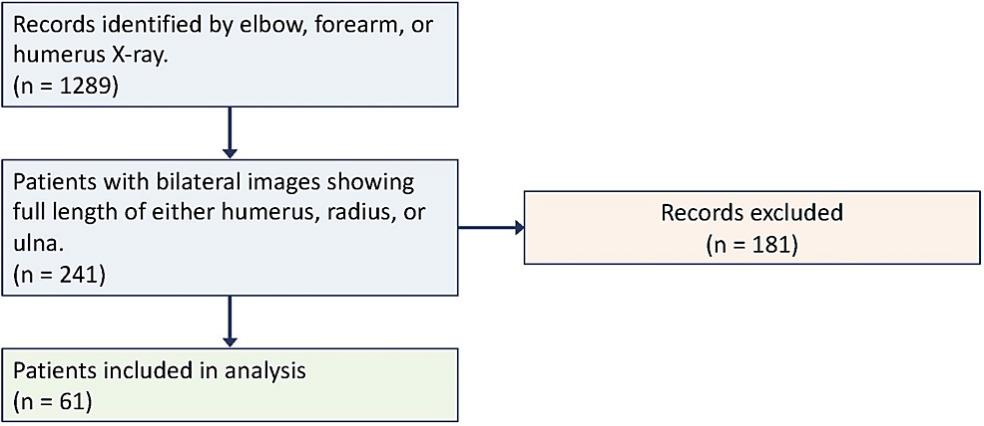
Inclusion and exclusion process flowchart.

Approval was obtained from the Texas Tech University Health Sciences Center (TTUHSC) Lubbock/Odessa Institutional Review Board (approval number: L20-060), and consent was waived as the study was retrospective and did not involve the collection or analysis of any identifiable information. This study was performed in accordance with the ethical standards laid down in the 1964 Declaration of Helsinki and its later amendments. Bilateral X-ray images (the elbow, forearm, or humerus) previously taken in a single orthopedic surgery department from years 2012-2019 for traumatic injury or chronic osseous conditions were collected for male and female adults from ages 18 to 81 (Figure 1). Subjects were excluded if they had a preexisting condition(s) known to affect bone length or if traumatic injury precluded length measurement of at least two long bones (the radius, ulna, or humerus). Only healthy bones were included for measurement and subsequent analysis. After screening, 61 patients met the inclusion criteria. Our cohort included 19 bilateral humerus, 28 radius, and 29 ulna measurements.

**Figure 2:**
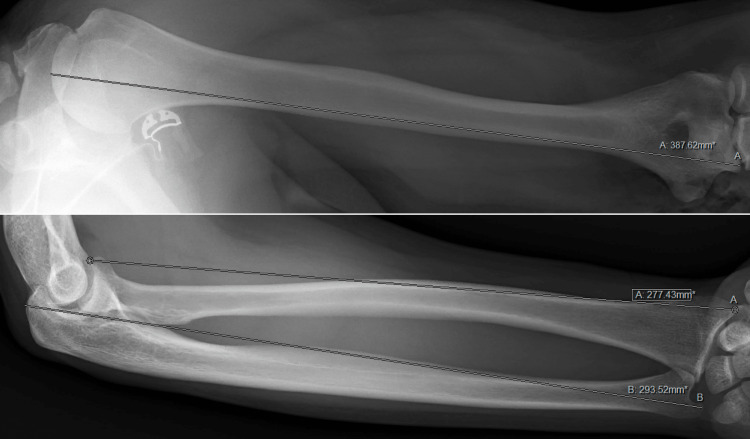
Measurement technique for humerus, radius, and ulna lengths.

Radius length was measured from the proximal head to the distal styloid process, ulna length was measured from the proximal olecranon process to the distal styloid process, and humerus length was measured from the proximal head to the distal trochlea (Figure 2). Three length measurements were taken by a single observer of each bone, and images were randomized to prevent biases caused by viewing previous measurements of the same bone [12]. In order to mitigate measurement inconsistency, the median length was taken from the three separate measurements and used for subsequent analysis (e.g., if patient number 1 had repeat ulna measurements of 293 mm, 294 mm, and 295 mm, the length used for analysis would be 294 mm).

Statistical analysis

**Figure 3:**
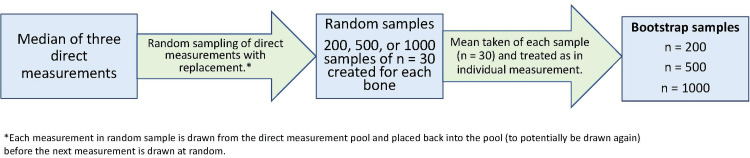
Bootstrapping method diagram.

Given the small sample of patients with bilateral imaging on a single date, a biostatistician was consulted, and bootstrapping (a random resampling process shown in Figure 3) was utilized to generate sample sizes (n) of 200, 500, and 1000 [13]. The Kolmogorov-Smirnov test of normality indicated that our data was normally distributed, and the paired t-tests were performed with our original measurements, as well as each bootstrap sample, to assess differences in contralateral bone lengths.

## Results

In our cohort, the mean absolute difference was 27.0 mm in the humerus, 8.6 mm in the radius, and 7.5 mm in the ulna (Table 1). None of the bones showed a trend toward a dominant (longer) side. In our cohort, 57.9% (n = 11/19) had right humerus measurements longer than left; in 60.7% (n = 17/28), the right radius was longer than the left; and in 48.3% (n = 14/29), the right ulna was longer than the left.

**Table 1:** Bootstrap samples: paired t-test results.

Bone	Total (n)	Mean absolute difference ± standard deviation	T score	P-value
Humerus	200	13.2 ± 10.3 mm	-5.817	<0.001
	500	14.0 ± 10.7 mm	-9.473	<0.001
	1000	14.1 ± 10.5 mm	-14.87	<0.001
Radius	200	16.3 ± 12.4 mm	2.258	0.025
	500	16.1 ± 12.04 mm	3.621	<0.001
	1000	16.0 ± 11.9 mm	5.496	<0.001
Ulna	200	15.2 ± 11.8 mm	1.907	0.058
	500	14.9 ± 11.6 mm	1.940	0.013
	1000	15.1 ± 12.0 mm	3.097	0.002

No significant differences between contralateral bones in any direct measurement group were found using Wilcoxon signed-rank tests (Table 1). Significant length differences were seen in all bootstrapped samples (n = 200, 500, and 1000) for both the humerus and radius but only the 500 and 1000 sample groups for the ulna (Table 2).

**Table 2:** Cohort measurements (direct sample): paired t-test results.

Bone	Total (n)	Mean absolute difference ± standard deviation	T score	P-value
Humerus	19	27.0 ± 24.5 mm	-0.954	0.353
Radius	28	8.6 ± 8.8 mm	1.587	0.124
Ulna	29	7.5 ± 8.1 mm	1.222	0.232

## Discussion

The results of our study indicate that a contralateral measurement may be adequate to estimate anatomical bone length when assessing length alterations in upper extremity long bones caused by congenital deformity, malignancy, or trauma. While the precise threshold has yet to be established, UE length alterations are thought to cause impaired normal motor function and/or pain with movement [4-6]. While multiple studies have addressed UE shortening, there is no consensus on the acceptable limit to retain maximal function [4-6]. In a prospective study of 22 proximal radial fractures, stable fractures with length alterations of less than 4 mm healed uneventfully and did not require operative treatment [4]. Another study showed that a shortening of 2 mm or greater in distal radius fractures resulted in greater disability at 6.5 years of follow-up [14].

In a report describing three cases of UE forearm lengthening through osteogenesis in the setting of congenital and/or pathological length discrepancy, researchers were able to demonstrate that an average of 2.3 cm of lengthening resulted in a 15-degree increase in forearm rotation [5]. In a separate study, 87.7% (79/90) of patients who underwent ulnar shortening osteotomy for ulnar-sided wrist pain reported “good or excellent” pain relief [6]. Such studies highlight the importance of understanding the degree of asymmetry in the UE, particularly in living populations, as length alterations can significantly impact function and pain [4-6].

While bootstrap samples achieved statistical significance for absolute difference between right and left lengths, there was no trend favoring greater length in either right or left UE long bones. Given that approximately 90% of the population is right-handed [15], one would expect the right UE to be significantly longer. However, this was not the case in our cohort. Studies of skeletal remains are often performed using pre-industrial humans, which are presumed to have been more physically active than the current population [2]. A large study of human remains found that industrial humans had less asymmetry in the upper extremities than pre-industrial humans from the same region [2]. A recent study of an agricultural community in North India showed a high degree of bilateral asymmetry in living males, further supporting the notion that modern, industrial humans may exhibit less asymmetry due to decreased physical activity [16].

The limitations of our study include a necessarily small sample size, the heterogeneity of our cohort, and the lack of standardized positioning for radiographs. True a priori power analysis was not performed. However, given the number of X-rays reviewed and those ultimately meeting the exclusion criteria (Figure 1), we estimate that approximately 9000 charts would need to be collected to obtain enough usable X-rays for statistical significance. These limitations were addressed by providing bootstrap samples and taking multiple bilateral measurements for each patient with a single observer.

## Conclusions

The current literature lacks bilateral length comparisons in living patients due to ethical constraints preventing bilateral radiographs for uninjured patients with no history of osseous disease. However, clinical decisions regarding the acceptable degree of length alteration require an accurate estimate of normal, pre-alteration length. The data we present may aid in the understanding of UE asymmetry in modern, living humans. While our findings do not indicate a need to adjust treatment algorithms, the results of this study may help to better define the role of asymmetry in length estimation.
